# Beyond multilocus barcoding: harnessing phylogenomics and genome mining for next-generation *Trichoderma*-based bioproducts

**DOI:** 10.1007/s00253-026-13834-w

**Published:** 2026-05-28

**Authors:** Nehal Atta, Abdelmegid I. Fahmi, Khalid S. Abdel-Lateif

**Affiliations:** Genetics Department, Faculty of Agriculture, Menoufia University, Shibin El-Kom, Egypt

**Keywords:** *Trichoderma*, Genomics, Multilocus identification, Strain improvement, Biocontrol, Biotechnology

## Abstract

**Abstract:**

*Trichoderma* species are among the most widely exploited fungal platforms in agricultural and industrial biotechnology due to their capacity to produce hydrolytic enzymes, secondary metabolites, and multifunctional biocontrol activities. However, their practical application has long been constrained by taxonomic complexity, strain-specific variability, and inconsistent field performance. Recent advances in molecular identification, multilocus sequence analysis, and genome-scale approaches have transformed *Trichoderma* research, enabling accurate species delimitation, functional trait prediction, and genome-guided strain selection. This review synthesizes how modern molecular and genomic tools bridge classical taxonomy with applied biotechnology in *Trichoderma*. Emphasis is placed on multilocus barcoding and phylogenomics as foundations for reliable strain identification, comparative and pan-genomic insights into enzymes and secondary metabolite biosynthetic clusters, and genetic improvement strategies including mutagenesis, protoplast fusion, and emerging genome-editing approaches. The review further highlights how these advances enhance biocontrol efficacy against fungal pathogens, plant-parasitic nematodes, and insect pests, while improving formulation stability and field consistency. By integrating taxonomy, genomics, and strain engineering, this review positions *Trichoderma* as a genomics-driven biotechnological platform and outlines key challenges and opportunities for its translation into reliable, sustainable bio-products.

**Key points:**

• *Multilocus and genome analyses enhance Trichoderma species and strain selection.*

• *Genome-guided insights link gene families to biocontrol and enzyme traits.*

• * Strain improvement enables effective biocontrol of fungi, nematodes, and pests.*

**Graphical Abstract:**

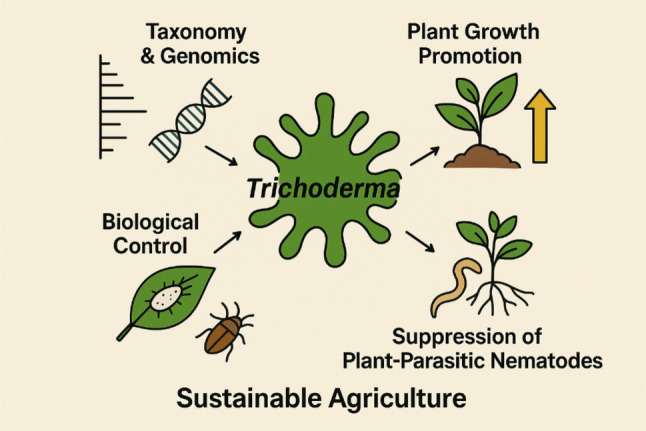

## Introduction

*Trichoderma* species represent one of the most extensively exploited fungal groups in modern agricultural and industrial biotechnology. Their success as biocontrol agents and biofactories is primarily attributed to rapid growth, metabolic versatility, secretion of hydrolytic enzymes, and the production of diverse secondary metabolites with antifungal, nematicidal, and insecticidal activities. Consequently, *Trichoderma* strains are widely incorporated into commercial formulations for crop protection, plant growth promotion, and enzyme production. Despite this broad utilization, practical applications remain constrained by pronounced strain-specific variability, inconsistent field performance, and challenges in reliably identifying strains (Guzmán-Guzmán et al. 2023).

Historically, *Trichoderma* taxonomy relied on morphological traits such as colony pigmentation, conidiophore architecture, and conidial morphology. While morphology remains useful for preliminary screening, phenotypic plasticity and convergence among closely related taxa have limited its reliability, particularly within species complexes such as *T. harzianum*, *T. asperellum*, and *T. viride*. Misidentification at the species or strain level has direct consequences for biotechnology, as genetically distinct isolates often differ markedly in enzymatic capacity, secondary metabolite profiles, stress tolerance, and antagonistic efficiency (Fahmi et al. 2016).

The integration of molecular tools has fundamentally transformed *Trichoderma* research. DNA barcoding using the ITS region enables rapid genus-level identification, while multilocus sequence analysis (MLSA), particularly employing tef1 and rpb2, provides higher resolution for accurate species delimitation. More recently, genome-scale approaches, including comparative genomics, phylogenomics, and pan-genomics, have revealed the genetic basis of key biotechnological traits, such as cell wall–degrading enzymes, secondary metabolite biosynthetic gene clusters, and mechanisms underlying mycoparasitism, induced systemic resistance, and stress adaptation. These advances have shifted *Trichoderma* research from descriptive systematics toward predictive, genome-guided strain selection (Naqvi et al. 2025).

Importantly, molecular and genomic insights are increasingly linked to applied outcomes. Accurate strain identification supports regulatory approval and quality control of commercial products, while genomic data enables the selection or improvement of elite strains through mutagenesis, protoplast fusion, and emerging genome-editing strategies. Such approaches have enhanced enzymatic activity, broadened the antagonistic spectrum, and improved performance against fungal pathogens, plant-parasitic nematodes, and insect pests. In this context, *Trichoderma* evolves from a classical biocontrol fungus into a multifunctional biotechnological platform (Woo et al. 2023).

This review focuses on advances in molecular identification, multilocus barcoding, and genomics, bridging taxonomy with applied biotechnology in *Trichoderma*. Emphasis is placed on genome-guided strain selection, genetic improvement strategies, and their translation into reliable biocontrol and biotechnological applications. By integrating systematics with functional genomics and strain engineering, this review highlights current challenges and future opportunities for optimizing *Trichoderma*-based products in sustainable biotechnology.

This review summarizes major advances in molecular and genomic tools applied to *Trichoderma* research during the last fifteen years, with particular emphasis on phylogenomics, comparative genomics, and genome-guided bioprospecting.

## Taxonomy as a foundation for applied strain selection

The genus *Trichoderma* was formally described by Tulasne in 1865, and early taxonomy relied primarily on morphological characters such as conidiophore branching patterns, phialide shape, conidial size and ornamentation, colony color and pigmentation, odor, and growth rates (Bissett 1984; Gams and Bissett 2002). These traits remain valuable for rapid and low-cost screening and often allow the differentiation of common species. However, morphological plasticity, environmental effects on colony appearance, and overlapping characters among closely related species, such as those in the *T. harzianum* and *T. viride* complexes, limit their discriminative power. Recent studies continue to demonstrate the utility of morphology, while highlighting its limitations; for example, 49 isolates from rhizosphere soils in India and several isolates from organic waste in Indonesia were initially grouped by colony color, topography, pigmentation, and spore size before molecular confirmation (Erayya et al. 2025; Muljowati et al. 2025). Over the past decade, polyphasic and molecular approaches, including multilocus sequencing (tef1, rpb2), ITS analysis, culture characteristics, secondary metabolite profiling, and rep-PCR, have become standard for resolving cryptic species, uncovering novel diversity, and linking genetic variability with functional traits such as cellulolytic potential (Zheng et al. 2021; Zhao et al. 2024; Hewedy et al. 2020; El-Sobky et al. 2024). Integrating morphological and molecular data thus provides a robust framework for accurate species identification and applied strain selection in biotechnology and sustainable agriculture.

Although multilocus barcoding using ITS, tef1, and rpb2 has significantly improved species identification, this approach still presents limitations in resolving closely related taxa and cryptic species complexes within the genus. For example, several species within the *Trichoderma harzianum* complex cannot be clearly differentiated using single- or multilocus markers due to high sequence similarity and possible recombination events. These limitations highlight the need for genome-scale phylogenetic approaches. Whole-genome sequencing now enables phylogenomic analyses based on hundreds or thousands of orthologous genes, providing higher resolution and redefining species boundaries within the genus Trichoderma (Rosolen et al. 2023).

Pan-genomic analyses have recently provided important insights into the genetic diversity of *Trichoderma*. In these studies, the genome is divided into a conserved core genome, shared by all strains of a species, and a variable accessory genome that contains strain-specific genes. The accessory genome often includes genes involved in secondary metabolism, environmental adaptation, and host interaction. This genomic variability may explain the frequently observed differences in biocontrol efficiency among strains belonging to the same species, particularly within the *Trichoderma harzianum* complex. Consequently, pan-genomics represents a powerful approach for identifying elite strains with superior biocontrol and biotechnological traits (Mondal et al. 2026).

## Ecology, habitat adaptation, and rhizosphere competence

*Trichoderma* species occupy diverse habitats, including soils, compost, decaying wood, rhizospheres, and plant tissues, and are also found in extreme or disturbed environments. For example, isolates from industrial wastewater in Pakistan revealed species not previously reported through morphological and molecular analyses (Bint-e-Zahira et al. 2024). In semiarid regions of Mexico and Latin America, species adapted to low moisture and high temperatures show distinct colony and spore traits, reflecting ecological adaptation (Rodríguez-Martínez et al. 2025). Environmental factors such as soil composition, metal stress, moisture, and nutrient availability influence colony pigmentation, sporulation, and aroma, which can complicate morphological identification. The ecological success of *Trichoderma* is supported by metabolic versatility and stress resilience, including expansions in gene families related to stress response, secondary metabolism, and plant interactions. In the rhizosphere, *Trichoderma* mobilizes nutrients via phosphate solubilization, organic acids, and siderophore-mediated iron chelation, interacts with root exudates, modulates microbial communities, and synergizes with beneficial microbes, enhancing nutrient acquisition, plant growth, and pathogen suppression under variable soil conditions (Woo et al. 2023; Song et al. 2024).

The metabolic activity of *Trichoderma* species is strongly influenced by environmental factors such as temperature, pH, nutrient availability, and interactions with plant hosts or competing microorganisms. These environmental conditions can regulate the expression of genes involved in secondary metabolism, enzyme production, and stress response. For example, nutrient limitation and plant root exudates can stimulate the production of hydrolytic enzymes and antimicrobial metabolites that contribute to the suppression of phytopathogens. Understanding the environmental regulation of metabolic pathways is therefore essential for improving the performance and consistency of *Trichoderma*-based biocontrol agents under field conditions (Ávila-Oviedo et al. 2026).

In addition to the well-known production of extracellular enzymes, *Trichoderma* species also possess highly active intracellular enzymatic systems involved in detoxification and xenobiotic metabolism. Among these systems, cytochrome P450 monooxygenases play a crucial role in the degradation and transformation of toxic compounds. These enzymes are capable of metabolizing a wide range of substrates, including plant defense compounds, environmental pollutants, and fungal toxins. The presence of diverse cytochrome P450 gene families in *Trichoderma* genomes highlights the adaptive capacity of these fungi to survive in complex ecological niches and contributes to their ability to colonize diverse environments (Chen et al. 2014).

## Molecular identification tools supporting biotechnology, including multilocus barcoding and phylogenomics

Traditionally, *Trichoderma* species have been identified based on morphological traits, including colony growth, pigmentation, odor, and microscopic features of conidiophores, phialides, and conidia (Bissett 1991a, b, c; Gams and Bissett 2002; Samuels et al. 2002). Colonies typically grow rapidly at 25–30 °C, producing green to bluish-green conidia, while some species, such as *T. viride*, emit a characteristic “coconut-like” odor. Microscopically, species are distinguished by highly branched conidiophores, flask-shaped phialides, smooth or roughened conidia, and chlamydospores that support survival under stress (Rex et al. 2001; Lin and Heitman 2005). Although these traits provide a useful framework for preliminary classification, morphological identification alone is often insufficient to differentiate cryptic or closely related species, highlighting the need for complementary molecular approaches for accurate species-level resolution.

The internal transcribed spacer (ITS) region of ribosomal DNA is the most widely used locus for fungal systematics and has been formally adopted as the universal DNA barcode for fungi (Schoch et al. 2012; Fahmi et al. 2016). In *Trichoderma*, ITS sequences enable rapid and cost-effective species identification and are routinely applied in biodiversity surveys, ecological studies, and biocontrol screening. ITS primers are straightforward to use, amplification success is generally high, and extensive reference sequences are available in public databases such as GenBank and UNITE, making ITS a logical first step in molecular identification. However, ITS has limited discriminatory power within cryptic species complexes, including *T. harzianum*, *T. koningii*, *T. asperellum*, and *T. viride*, where closely related taxa often share identical or nearly identical haplotypes (Druzhinina et al. 2005; Jaklitsch and Voglmayr 2015). For example, re-examination of 148 strains labeled as *T. harzianum* showed that ITS alone misclassified many isolates, which were correctly resolved using multilocus phylogenies with tef1α and rpb2 (Ismaiel et al. 2024a, b). Similar patterns were observed in Rajasthan and Egyptian isolates, where ITS provided broad clustering but failed to capture intra-species variation, whereas multilocus approaches revealed higher resolution and enabled discovery of novel species (Meena and Jambhulkar 2024; El-Sharkawy et al. 2024; Sun et al. 2025). Overall, ITS remains a valuable primary barcode for preliminary identification, but accurate species-level resolution in *Trichoderma* requires multilocus phylogenetic analysis.

To overcome the limited resolution of the ITS barcode, several protein-coding genes have been increasingly used for *Trichoderma* taxonomy and identification. Among these, the translation elongation factor 1-alpha (tef1) gene has emerged as the most informative locus, providing strong phylogenetic signal and resolving species boundaries within complexes such as *T. harzianum*, *T. asperellum*, and *T. koningii* (Druzhinina et al. 2005; Jaklitsch and Voglmayr 2015). Recent multilocus studies confirm its reliability; for example, tef1 combined with ITS distinguished closely related isolates from Indian soils that clustered together with ITS alone (Meena and Jambhulkar 2024). The RNA polymerase II subunit B gene (rpb2) is another highly informative marker, particularly for cryptic or newly described taxa, and has higher resolution than ITS. For instance, isolates from Yunnan karst soils were separated into novel species only after rpb2 was included alongside ITS and tef1 (Sun et al. 2025). Other loci, including act1 (actin), cal1 (calmodulin), and tub2 (beta-tubulin), are used as supplementary markers, adding phylogenetic robustness in challenging complexes (Błaszczyk et al. 2021). Collectively, multilocus sequence analysis (MLSA), especially combining ITS, tef1, and rpb2, is now considered the gold standard for *Trichoderma* species delimitation, enhancing taxonomic accuracy and providing a solid framework for applied research, including biocontrol strain selection and regulation (Table 1) (Guzmán-Guzmán et al. 2024; Pfordt et al. 2025

**Table 1:** Common molecular markers used for *Trichoderma* identification and their applications

Marker	Function/target	Advantages	Limitations	Key references
ITS (internal transcribed spacer)	Universal fungal barcode (rDNA region)	Rapid identification, widely available in databases, good for genus-level ID	Limited resolution in species complexes (*T. harzianum*, *T. koningii*)	Druzhinina et al. 2005; Sharma et al. 2023
tef1 (translation elongation factor 1-α)	Protein-coding gene involved in translation	High resolution across species complexes, widely considered most reliable marker	Requires high-quality sequencing	Jaklitsch and Voglmayr 2015; Meena and Jambhulkar 2024
rpb2 (RNA polymerase II subunit B)	Encodes catalytic subunit of RNA polymerase II	Excellent for distinguishing cryptic and novel species, strong phylogenetic signal	Fewer reference sequences than ITS	Sun et al. 2025
act1 (actin)	Cytoskeletal protein	Useful in multilocus phylogenies, adds robustness	Limited resolution when used alone	Błaszczyk et al. 2021
cal1 (calmodulin)	Calcium-binding protein	Supplementary marker, supports multilocus trees	Less discriminatory individually	Guzmán-Guzmán et al. 2024
tub2 (β-tubulin)	Structural protein in microtubules	Occasionally informative, useful for some groups	Inconsistent resolution across genus	Pfordt et al. 2025
MLSA (multilocus sequence analysis: ITS + tef1 + rpb2)	Combined approach for taxonomy	Gold standard for accurate species delimitation, robust in resolving complexes	Requires multiple gene sequencing	Guzmán-Guzmán et al. 2024; Pfordt et al. 2025

With the advent of next-generation sequencing (NGS), genome-scale data are increasingly applied to the taxonomy and phylogeny of *Trichoderma*. Whole-genome sequencing and comparative genomics enable the analysis of hundreds to thousands of orthologous genes, providing unprecedented phylogenetic resolution and facilitating the redefinition of species boundaries. These phylogenomic approaches clarify relationships within problematic species complexes and reveal evolutionary dynamics, including horizontal gene transfer, genome plasticity, and ecological adaptation. Genome-scale studies also illuminate the genetic basis of key traits, such as mycoparasitism, secondary metabolite production, and plant-beneficial functions, including induction of systemic resistance and nutrient acquisition. Advances in pan-genomics and population genomics further uncover intra-species diversity, which is critical for the selection of elite strains for biocontrol and agricultural applications. Publicly available genomic resources, including Ensembl Fungi, MycoCosm, and NCBI Genome, provide essential platforms for integrative taxonomy, functional annotation, and comparative analyses (Table 2). As sequencing technologies become more cost-effective, genome-based approaches are expected to complement, and ultimately supersede, traditional multilocus methods, establishing a new standard for *Trichoderma* systematics (Naqvi et al. 2025).

**Table 2:** Genomic resources and their applications in *Trichoderma* research

Genomic resource/tool	Description	Applications in *Trichoderma*	References
Whole-genome sequencing (WGS)	High-throughput sequencing of entire genomes	Species delimitation, discovery of cryptic diversity, and evolutionary studies	Prismantoro et al. 2025
Comparative genomics	Cross-species genome comparisons	Identification of gene clusters for mycoparasitism, secondary metabolism, and stress tolerance	Sun et al. 2025
Phylogenomics	Orthologous gene-based phylogenetic analysis	Resolving species complexes (e.g., *T. harzianum*, and *T. asperellum*)	Pfordt et al. 2025
Pan-genomics	Analysis of core and accessory genomes	Understanding strain-level variation, adaptation, and biocontrol potential	Pfordt et al. 2025
Population genomics	Genome-wide variation across populations	Insights into domestication, biogeography, and selection pressures	Prismantoro et al. 2025
Databases (Ensembl Fungi, MycoCosm, NCBI Genome)	Curated genomic repositories	Access to annotated genomes for systematics and functional studies	Public repositories

## Genomic resources and functional trait prediction through comparative and pan-genomic analyses

The rapid expansion of genomic resources has fundamentally reshaped *Trichoderma* research, transforming strain selection from empirical screening into a predictive, genome-guided process (Schalamun and Schmoll 2022; Guzmán-Guzmán et al. 2025). Whole-genome sequencing (WGS) provides high-resolution insight into species boundaries, evolutionary relationships, and intraspecific diversity, while comparative genomics and phylogenomics resolve cryptic species complexes and clarify functional divergence among closely related taxa (Jaklitsch and Voglmayr 2015; Pfordt et al. 2025; Prismantoro et al. 2025).

Beyond taxonomy, genome-scale analyses reveal the genetic architecture underlying key biotechnological traits. Highly effective *Trichoderma* strains are characterized by expanded gene families encoding cell wall–degrading enzymes, transporters, regulatory proteins, and stress-response factors, as well as a rich repertoire of secondary metabolite biosynthetic gene clusters (Sood et al. 2020; Schalamun and Schmoll 2022; Sun et al. 2025). Comparative and pan-genomic studies demonstrate that variation within accessory genomes largely explains strain-specific differences in antagonistic efficiency, rhizosphere competence, and environmental resilience (Pfordt et al. 2025; Ismaiel et al. 2024a, b).

Importantly, genomic data now enable functional trait prediction prior to extensive phenotypic evaluation. The presence, diversity, and organization of genes associated with mycoparasitism, secondary metabolism, and plant interaction can be used to identify elite strains with high biocontrol potential and broad ecological adaptability (Sood et al. 2020; Guzmán-Guzmán et al. 2024). Population genomic approaches further provide insight into domestication, local adaptation, and selection pressures, informing rational strain deployment across contrasting agro-ecological environments (Ismaiel et al. 2024a, b; Prismantoro et al. 2025).

Public genomic repositories, including Ensembl Fungi, MycoCosm, and the NCBI Genome database, facilitate integrative analyses by offering curated and annotated *Trichoderma* genomes for comparative and functional studies (Schalamun and Schmoll 2022). As sequencing technologies continue to advance and costs decline, genome-based prediction of biocontrol performance is expected to complement—and in some cases supersede—traditional multilocus identification and trial-and-error screening, accelerating the development of reliable and consistent *Trichoderma*-based biotechnological products (Guzmán-Guzmán et al. 2025; Pfordt et al. 2025).

### Genome mining

Genome mining has become a powerful strategy for discovering novel secondary metabolites in *Trichoderma*. Bioinformatic pipelines such as antiSMASH, BiG-SCAPE, and DeepBGC enable the systematic identification and classification of biosynthetic gene clusters (BGCs) in fungal genomes. These tools allow researchers to detect cryptic or silent BGCs that are not expressed under standard laboratory conditions. Through comparative genomic analyses and cluster prediction, genome mining can reveal previously unknown metabolites with potential antifungal, antibacterial, or plant-growth-promoting activities. Such approaches greatly expand the potential of *Trichoderma* as a source of novel bioactive compounds (Zhang et al. 2025).

### Systems biology (WGCNA)

Systems biology approaches integrating transcriptomics and gene co-expression analyses are increasingly applied to understand the functional regulation of *Trichoderma* genomes. Methods such as weighted gene coexpression network analysis (WGCNA) allow the identification of gene modules associated with stress tolerance, energy metabolism, and host interaction. These approaches help bridge the gap between genomic potential and the actual phenotypic performance of *Trichoderma* strains under field conditions (Geddes-McAlister 2022).

Systems biology approaches are increasingly used to understand the functional regulation of *Trichoderma* genomes. Integration of transcriptomic data, particularly RNA-seq analysis, allows researchers to investigate how gene expression changes under different environmental conditions such as abiotic stress, nutrient limitation, or plant interaction. In addition, gene coexpression network analyses using methods such as weighted gene coexpression network analysis (WGCNA) enable the identification of gene modules associated with specific biological processes. These approaches help reveal regulatory networks involved in energy metabolism, stress tolerance, and secondary metabolite production. By linking genomic potential with actual gene expression patterns, systems biology provides a deeper understanding of the mechanisms underlying the ecological fitness and biocontrol performance of *Trichoderma* strains (Rosolen et al. 2022).

### Transcriptomics

Proteomic approaches have become increasingly important for understanding the functional biology of *Trichoderma*. While genomic analyses reveal the genetic potential of an organism, proteomics provides direct evidence of which proteins are actually produced under specific environmental conditions. Mass spectrometry–based proteomic analyses have been used to identify enzymes involved in mycoparasitism, plant interaction, and secondary metabolism. These studies have revealed the expression of numerous hydrolytic enzymes, transport proteins, and stress-response factors that contribute to the ecological fitness of *Trichoderma*. Consequently, integrating genomics with proteomics provides a more comprehensive understanding of the molecular mechanisms underlying biocontrol activity (Druzhinina et al. 2011).

## Biocontrol mechanisms with biotechnological relevance

*Trichoderma* species exhibit a multifaceted biocontrol strategy that integrates direct antagonism of plant pathogens with indirect, plant-mediated defense responses. These mechanisms are not independent but operate synergistically, forming a robust functional platform that underpins the broad biotechnological applicability of *Trichoderma* in agriculture and beyond (Harman et al. 2004; Sood et al. 2020; Monte 2023).

Mycoparasitism represents a hallmark biocontrol mechanism of *Trichoderma*, involving host recognition, hyphal attachment and coiling, penetration, and subsequent degradation of target fungal structures (Benítez et al. 2004; Harman et al. 2004). This process is mediated by the coordinated secretion of cell wall–degrading enzymes (CWDEs), including chitinases, β−1,3-glucanases, and proteases, which collectively dismantle pathogen cell walls and compromise viability (Kubicek et al. 2011; Druzhinina et al. 2018). Genomic analyses reveal that many effective biocontrol strains harbor expanded CWDE gene families, supporting the strong link between enzymatic capacity and antagonistic performance (Schalamun and Schmoll 2022).

In addition to enzymatic degradation, *Trichoderma* produces a diverse array of secondary metabolites that inhibit pathogen growth and development. These include peptaibols, gliotoxin, viridin, pyrones, and volatile organic compounds, which act through membrane disruption, oxidative stress induction, and interference with cellular signaling pathways (Mukherjee et al. 2012; Sood et al. 2020; Monte 2023). Advances in genome mining have identified numerous biosynthetic gene clusters associated with these metabolites, underscoring the biotechnological potential of *Trichoderma* as a source of novel bioactive compounds (Kubicek et al. 2011; Schalamun and Schmoll 2022).

Rapid growth, efficient nutrient utilization, and strong rhizosphere competence further enhance the biocontrol efficacy of *Trichoderma*. These traits enable competitive exclusion of phytopathogens through superior colonization of root surfaces and depletion of available resources (Harman et al. 2004; Druzhinina et al. 2018). Genomic features such as expanded transporter families and regulatory networks contribute to environmental adaptability and persistence under fluctuating soil conditions (Pfordt et al. 2025).

Beyond direct antagonism, *Trichoderma* interacts intimately with host plants, eliciting induced systemic resistance (ISR) that enhances plant defenses against a broad range of biotic stresses. Root colonization activates jasmonic acid- and salicylic acid-dependent signaling pathways, leading to primed defense responses and reduced susceptibility to pathogens (Shoresh et al. 2010; Sood et al. 2020). These plant-mediated effects not only contribute to disease suppression but also improve plant vigor and stress tolerance, reinforcing the value of *Trichoderma* as a multifunctional biotechnological agent (Harman et al. 2021; Monte 2023).

Genomic information is also becoming increasingly important in the regulatory approval of microbial biocontrol agents. Whole-genome analysis can confirm the absence of genes associated with mycotoxin production or other undesirable traits, providing evidence of biosafety for commercial registration. Consequently, genome-based safety assessment is emerging as a key requirement for the development and regulatory acceptance of new *Trichoderma*-based biofungicides (Kulik et al. 2025).

Secondary metabolites produced by *Trichoderma* can directly interfere with the metabolism of phytopathogenic fungi. These compounds may inhibit key cellular processes such as cell wall synthesis, mitochondrial respiration, and membrane integrity. In some cases, metabolites produced by *Trichoderma* disrupt signal transduction pathways in phytopathogens, reducing their virulence and ability to colonize plant tissues. Additionally, certain metabolites can induce oxidative stress in pathogenic fungi, leading to impaired growth and cell damage. These metabolic interactions play a central role in the antagonistic activity of *Trichoderma* and contribute to its effectiveness as a biological control agent (Khan et al. 2020).

## Translation of mechanisms into applications against fungal pathogens, nematodes, and insect pests

*Trichoderma* species are effective against a broad range of soilborne pathogens through multiple modes of action (Table 3). *Rhizoctonia solani*, a necrotrophic pathogen causing damping-off, root rot, and stem canker in crops such as cotton, tomato, and legumes, is suppressed by *T. harzianum*, *T. viride*, and *T. asperellum* via hyphal parasitism, sclerotial degradation, and production of inhibitory volatiles like 6-pentyl-α-pyrone; field trials have reported yield increases of 25–30% following seed treatment or soil amendment (Hasna et al. 2025). *Macrophomina phaseolina*, the charcoal rot pathogen affecting soybean, sesame, and sorghum under drought, is controlled by *T. virens* and *T. asperellum* through antifungal volatiles, CWDE secretion, and enhanced plant defense enzyme activity, achieving 60–70% disease reduction under field conditions (Ayaz et al. 2023). *Fusarium oxysporum*, responsible for vascular wilts, is suppressed by *T. asperellum* and *T. harzianum*, which inhibit growth via antibiosis and induce systemic resistance; genomic analyses show that effective strains often possess expanded chitinase gene families and secondary metabolite clusters (Schalamun and Schmoll 2022). *Pythium* spp., causal agents of damping-off in cereals and vegetables, are controlled by *T. viride* and *T. atroviride* through rapid root colonization, direct inhibition, and competitive exclusion, enhancing seedling establishment (Sultana et al. 2023). *Sclerotium rolfsii*, causing southern blight and collar rot, is effectively suppressed by *T. harzianum* via sclerotia parasitism and antifungal metabolite production. Omics-based studies reveal that strain-specific differences in secondary metabolite and CWDE gene clusters largely determine pathogen-specific activity, emphasizing the importance of careful strain selection for field applications (Sun et al. 2025).

**Table 3:** Major soilborne pathogens controlled by *Trichoderma*

Pathogen	Crops affected	Effective *Trichoderma* spp.	Mode(s) of action	Recent references
*Rhizoctonia solani*	Tomato, cotton, beans, cereals	*T. harzianum*, *T. viride*, *T. asperellum*	Hyphal coiling, sclerotial degradation (CWDEs), volatile metabolites (6-pentyl-α-pyrone), ISR	Hasna et al. 2025; Gutiérrez-Moreno et al. 2025a, b
*Macrophomina phaseolina*	Soybean, sesame, sorghum	*T. virens*, *T. asperellum*	Antifungal volatiles, chitinases, phenylalanine ammonia lyase induction, ISR	Ayaz et al. 2023
*Fusarium oxysporum* (wilt complex)	Tomato, banana, legumes, cotton, chickpea	*T. asperellum*, *T. harzianum*, *T. viride*	Competition, secondary metabolites (gliotoxin, harzianopyridone), ISR	Kumari et al. 2024; Sun et al. 2025
*Pythium *spp.	Vegetables, cereals, ornamentals	*T. viride*, *T. atroviride*	Rapid root colonization, nutrient competition, secretion of glucanases, seedling vigor enhancement	Hasna et al. 2025
*Sclerotium rolfsii*	Groundnut, legumes, vegetables	*T. harzianum*, *T. virens*	Sclerotial parasitism, antifungal metabolites, mycoparasitism	Ayaz et al. 2023; Gutiérrez-Moreno et al. 2025a, b

Plant-parasitic nematodes, particularly root-knot nematodes (*Meloidogyne* spp.), are among the most destructive pathogens in global agriculture, causing estimated annual yield losses of 12–20%. Conventional nematicides are often costly, environmentally hazardous, and increasingly restricted by regulations, highlighting the need for sustainable alternatives. *Trichoderma* species have emerged as effective biocontrol agents against nematodes, acting through both direct parasitism of nematode eggs and juveniles and the induction of plant-mediated defense mechanisms, including systemic resistance and enhanced expression of defense-related enzymes (Table 4) (Contreras-Soto et al. 2025).

**Table 4:** Plant-parasitic nematodes targeted by *Trichoderma* spp. and their biocontrol mechanisms

Nematode pest	Host crop/system	Effective *Trichoderma* spp.	Mode of action	References
*Meloidogyne incognita* (root-knot nematode)	Tomato, cucumber, legumes	*T. harzianum*, *T. asperellum*, *T. viride*	Egg parasitism, protease/chitinase activity, inhibition of hatching, induced resistance	Uddin et al. 2023; Baidya et al. 2023
*Meloidogyne javanica*	Tomato, eggplant	*T. harzianum* T22, *T. viride*	Reduced gall formation, JA/SA-mediated systemic resistance, plant growth promotion	Contreras-Soto et al. 2025; Solano-Baidya et al. 2023
*Meloidogyne arenaria*	Soybean, vegetables	*T. asperellum*	Antibiosis, lytic enzyme secretion, suppression of juvenile penetration	Adomako et al. 2025
Mixed *Meloidogyne* spp.	Tomato, field crops	*T. harzianum* + organic amendments/*Pasteuria penetrans*	Synergistic suppression, reduced egg masses, enhanced crop vigor	El-Nagdi et al. 2019; Saleh et al. 2023

Several *Trichoderma* species, including *T. harzianum*, *T. asperellum*, and *T. viride*, produce lytic enzymes such as chitinases, proteases, and glucanases, along with toxic secondary metabolites that degrade nematode egg shells and juvenile cuticles, reducing hatchability and increasing juvenile mortality (Baidya et al. 2023). In vitro assays report up to 80% inhibition of eggs and 82% juvenile mortality of *Meloidogyne incognita* by *T. harzianum* isolates (Uddin et al. 2023). Greenhouse studies further confirm that plants treated with these isolates exhibit significantly fewer galls and egg masses, demonstrating the practical efficacy of enzymatic and metabolite-mediated nematode suppression (Rostami et al. 2024; Adomako et al. 2025).

Beyond direct antagonism, *Trichoderma* enhances host plant resistance through induced systemic resistance (ISR). Root colonization activates defense pathways mediated by jasmonic acid (JA) and salicylic acid (SA), reducing nematode invasion and reproduction (Contreras-Soto et al. 2025). These plant-mediated effects often translate into improved growth and yield, mitigating nematode-induced stunting. For instance, inoculation with *T. harzianum* T22 significantly reduced gall formation in tomato and enhanced biomass accumulation, even under heavy *Meloidogyne javanica* infection (Baidya et al. 2023).

Recent studies emphasize the potential of *Trichoderma* in integrated management strategies for plant-parasitic nematodes. For example, combining *T. harzianum* with organic amendments and reduced doses of nematicides provided superior suppression of *Meloidogyne incognita* compared to individual treatments (El-Nagdi et al. 2019). Similarly, *T. harzianum* applied alongside the bacterial parasite *Pasteuria penetrans* enhanced control of *M. javanica* in tomato (Saleh et al. 2023). These findings highlight the value of *Trichoderma* in synergistic formulations that integrate microbial and chemical approaches, offering more effective and sustainable nematode management.

The accumulated evidence demonstrates that *Trichoderma* spp. are reliable biocontrol agents against plant-parasitic nematodes, acting through multiple mechanisms including parasitism, antibiosis, competition, and induced systemic resistance. However, efficacy is strain-dependent and influenced by soil type, crop species, and environmental conditions. Future research should prioritize genome-assisted selection of nematophagous strains, development of formulations that enhance field persistence, and integration of *Trichoderma* into broader integrated pest management (IPM) programs to maximize sustainable nematode control (Contreras-Soto et al. 2025).

Although *Trichoderma* is best known for its antagonism against phytopathogenic fungi, accumulating evidence demonstrates that several species also possess entomopathogenic traits, expanding their role in integrated pest management (IPM). Laboratory and greenhouse studies show that certain *Trichoderma* strains can cause significant mortality in economically important insect pests across multiple orders, including Lepidoptera, Coleoptera, Hemiptera, and Diptera (Table 5). Direct mechanisms involve penetration of the insect cuticle, facilitated by hydrolytic enzymes such as chitinases, proteases, and lipases, followed by colonization of internal tissues. Secondary metabolites, including peptaibols, gliotoxin, and viridin, exert toxic effects that impair insect survival, growth, and reproduction. Indirectly, *Trichoderma* can prime host plant defense pathways, reducing herbivore performance (Chen et al. 2025).

**Table 5:** Insect pests targeted by *Trichoderma* spp., their biocontrol mechanisms, and type of effect

Insect pest	Host crop/system	Effective *Trichoderma* spp.	Mode of action	Type of effect	References
*Spodoptera littoralis* (cotton leafworm)	Cotton, vegetables	*T. harzianum*, *T. asperellum*	Cuticle penetration, protease/chitinase activity, secondary metabolites	Direct	Hasna et al. 2025
*Sitotroga cerealella* (angoumois grain moth)	Stored cereals	*T. viride*, *T. harzianum*	Spore adhesion, enzymatic degradation, mycotoxin production	Direct	Chen et al. 2025
*Callosobruchus maculatus* (cowpea bruchid)	Stored legumes	*T. viride*, *T. asperellum*	Mycelial overgrowth, toxin production	Direct	Ayaz et al. 2023
*Rhynchophorus ferrugineus* (red palm weevil)	Date palm	*T. harzianum*	Direct pathogenicity, enzyme-mediated cuticle degradation	Direct	Hasan et al. 2025
*Nezara viridula* (southern green stinkbug)	Sweet pepper	*T. harzianum* T22	Induced systemic resistance, alteration of host plant metabolites	Indirect (plant-mediated)	Van Hee al. 2025
*Myzus persicae* (green peach aphid)	Pepper, vegetables	*T. harzianum*, *T. viride*	Plant-mediated resistance, reduced fecundity	Indirect (plant-mediated)	Kara et al. 2025

Notable examples include: *Spodoptera littoralis* (cotton leafworm), where *T. harzianum* and *T. viride* strains caused significant larval mortality and growth inhibition (Ahmed and El-Katatny 2007; Lana et al. 2023; Di Lelio et al. 2021; Atta et al. 2025); *Rhynchophorus ferrugineus* (red palm weevil), suppressed by *T. harzianum* through reduced adult survival and feeding (Hasan et al. 2025); *Nezara viridula* (southern green stink bug), whose performance decreased on pepper plants treated with *T. harzianum* T22 via induced systemic resistance (Van Hee et al. 2025); and *Myzus persicae* (green peach aphid), where reproductive performance was reduced on plants inoculated with *T. harzianum* and *T. viride* (Kara 2025).

Recent experimental work further supports these entomopathogenic activities. Egyptian isolates of *T. asperellum*, *T. harzianum*, and *T. longibrachiatum* exhibited high chitinase activity and potent insecticidal effects against *S. littoralis* larvae, with oral spore applications causing higher mortality than topical treatments, highlighting the synergistic roles of hydrolytic enzymes and secondary metabolites (Atta et al. 2025). Although *Trichoderma* is not yet a primary entomopathogen compared with fungi such as *Beauveria bassiana* and *Metarhizium anisopliae*, its dual capacity to suppress both pathogens and insect pests positions it as a valuable complementary tool in IPM programs (Chen et al. 2025; Hasna et al. 2025). Advances in molecular and genomic analyses are expected to accelerate exploration of *Trichoderma*’s insect-interaction pathways, enhancing its potential as a multifunctional biocontrol agent.

In addition to direct antagonism, *Trichoderma* triggers complex signaling cascades in plants that prime immunity. Root recognition of fungal microbe-associated molecular patterns (MAMPs) and damage-associated molecular patterns (DAMPs) activates both salicylic acid (SA) and jasmonic acid/ethylene (JA/ET) pathways, establishing a heightened defensive state that allows plants to respond more rapidly and effectively to subsequent pathogen attacks. This priming does not impose a continuous metabolic cost but creates a transcriptional “memory,” which can persist across generations, a phenomenon known as heritable priming. Colonization by *T. harzianum* or *T. asperellum* has been shown to upregulate pathogenesis-related proteins, peroxidases, and phenylpropanoid enzymes, enhancing disease resistance and tolerance to abiotic stresses. These systemic effects position *Trichoderma* as a unique biocontrol agent, bridging defense activation with growth promotion (Woo et al. 2023; Poveda et al. 2021; Monte 2023).

## Genome-guided strain improvement strategies

Genetic improvement strategies have long been applied to enhance the biocontrol and enzymatic performance of *Trichoderma* strains. Early approaches, including random mutagenesis (Marzano et al. 2013) and protoplast fusion (Prabavathy et al. 2006), yielded *T. harzianum* isolates with enhanced enzymatic activity and stronger antagonism against pathogens. Advances in understanding the molecular basis of biocontrol (Sood et al. 2020; Adnan et al. 2022) have enabled targeted genetic engineering to improve strain efficiency (Chen et al. 2021); however, most commercial products still rely on wild-type isolates due to technical limitations and regulatory or public concerns regarding genetically modified organisms (Xiao et al. 2023).

Protoplast fusion offers a nontransgenic alternative for filamentous fungi lacking sexual reproduction, allowing genome recombination between compatible *Trichoderma* strains to generate hybrids with enhanced enzyme secretion, pathogen inhibition, and stress tolerance (Peberdy 1980). Fusants have demonstrated increased cellulase, chitinase, and xylanase activities, along with improved antagonistic potential (Ogawa et al. 1989; Fahmi et al. 2012; Dolatabad et al. 2019; Papzan et al. 2021). Recent studies have extended these approaches to pest control: Atta et al. (2025) fused *T. asperellum* and *T. harzianum* isolates to generate twenty stable hybrids, with the most effective, Fus8, exhibiting significantly enhanced chitinase activity and larvicidal efficacy against *Spodoptera littoralis*, achieving 74% mortality in laboratory assays and 62% under greenhouse conditions. These results demonstrate that parasexual recombination can produce elite, eco-friendly *Trichoderma* strains with dual biofungicidal and bioinsecticidal potential.

## Field performance, formulation, and regulatory constraints

The commercial use of *Trichoderma* as a biocontrol agent is well established, with multiple formulations registered worldwide. These products are applied as seed treatments, soil amendments, and foliar sprays to protect crops such as maize, tomato, cotton, and legumes. *Trichoderma*-based products offer several advantages, including broad-spectrum activity against pathogens and pests, promotion of plant growth and stress tolerance, and compatibility with sustainable and organic farming practices.

However, several challenges limit their widespread adoption (Table 6. Strain variability significantly affects efficacy, with notable differences even among isolates of the same species. Environmental factors, such as soil type, climate, and native microbial communities, can cause inconsistent field performance. Formulation issues, including maintaining viability, shelf-life, and field persistence, remain technological bottlenecks. Additionally, regulatory constraints vary across countries, slowing the commercialization and deployment of new products. Addressing these challenges is essential to fully exploit the potential of *Trichoderma* in sustainable agriculture (Gutiérrez-Moreno et al. 2025a, b).

**Table 6:** Advantages and challenges of *Trichoderma *-based biocontrol

Aspect	Advantages	Challenges	References
Spectrum of action	Controls fungi (*Fusarium*, *Rhizoctonia*, *Pythium*), nematodes, and some insects	Strain-specific variability; inconsistent results across isolates	Ayaz et al. 2023; Hasna et al. 2025
Environmental impact	Eco-friendly, biodegradable, compatible with organic farming	Field performance often unpredictable due to soil, climate, and microbiome interactions	Guzmán‑Guzmán et al. 2025
Plant growth effects	Promotes root development, nutrient uptake, and induces systemic resistance	Overstimulation or imbalance may cause variability in yield outcomes	Guzmán-Guzmán et al. 2025
Formulation	Available in diverse delivery forms (seed coating, soil drench, foliar spray, granules)	Shelf-life, storage stability, and viability during transport remain bottlenecks	Ismaiel et al. 2024
Commercialization	Many registered products worldwide; integration into IPM programs	Regulatory hurdles differ by country; approval can be lengthy and costly	Hasna et al. 2025

Globally, over 140 *Trichoderma*-based biocontrol products are registered across more than 40 countries, applied as seed treatments, soil inoculants, and foliar sprays. Regulatory classification varies widely: some countries treat these products as plant protection products (PPPs), while others categorize them as biostimulants. A unified framework recognizing *Trichoderma* as a plant-beneficial microorganism is essential to harmonize approval procedures and facilitate innovation.

Future formulation strategies increasingly emphasize microbial consortia, combining *Trichoderma* with plant growth-promoting rhizobacteria (PGPR) and arbuscular mycorrhizal fungi (AMF), as well as advanced delivery systems such as nanocarriers to enhance shelf life, stress tolerance, and root colonization efficiency (Woo et al. 2023; Hasna et al. 2025; Pfordt et al. 2025). These approaches aim to maximize field performance and support the integration of *Trichoderma* into sustainable agricultural practices.

## Future perspectives toward genomics-driven *Trichoderma* bio-products

The integration of classical biocontrol knowledge with modern molecular tools presents promising avenues for advancing *Trichoderma* applications. Key directions include the following:

- Genomics-driven taxonomy—refining species concepts and ensuring accurate identification of biocontrol strains.
- Strain improvement via omics and genome editing—enhancing enzyme secretion, secondary metabolite production, and stress tolerance.
- Formulation innovations—development of nanocarriers and stress-protective additives to improve field stability and efficacy.
- Climate-smart agriculture—exploiting *Trichoderma*’s capacity to mitigate abiotic stresses such as drought and salinity in addition to disease suppression.
- Expanded applications—leveraging *Trichoderma* in biofertilizers, phytoremediation, and industrial enzyme production.

Genomics-driven taxonomy and comparative genomics (Guzmán-Guzmán et al. 2025) are expected to refine strain identification and improve the selection of elite isolates. Genome-guided strain improvement and CRISPR-based approaches are increasingly used to optimize metabolite biosynthesis, enzyme secretion, and environmental resilience (Ismaiel et al. 2024a, b). Integrating *Trichoderma* with other beneficial microbes, such as plant growth-promoting rhizobacteria (PGPR) and arbuscular mycorrhizal fungi (AMF), may further enhance field performance (Hasna et al. 2025).

Next-generation *Trichoderma*-based products are envisioned to combine multiple functionalities—biocontrol, growth promotion, nutrient mobilization, and stress tolerance—within a single, stable formulation. Advances in pan-genomics, metabolomics, and genome editing will facilitate the selection of elite strains with predictable performance under field conditions. Moreover, *Trichoderma*’s contributions to carbon sequestration, soil health restoration, and climate-resilient cropping systems underscore its potential as a cornerstone of eco-sustainable agriculture and One Health strategies (Woo et al. 2023; Guzmán-Guzmán et al. 2025).

## Conclusions

Advances in molecular identification and genomics have fundamentally reshaped the understanding and utilization of Trichoderma in applied microbiology and biotechnology. While classical morphology and ITS barcoding remain valuable for preliminary classification, multilocus sequencing and genome-scale analyses are now essential for accurate species delimitation and reliable strain selection. These tools have clarified cryptic species boundaries and revealed extensive genetic diversity underlying strain-specific differences in enzymatic activity, secondary metabolism, and biocontrol efficacy.

Genomic and comparative studies demonstrate that the biotechnological potential of *Trichoderma* is largely determined by expansions in gene families encoding cell wall–degrading enzymes, secondary metabolite biosynthetic clusters, and regulatory networks involved in stress tolerance and host interaction. Such insights enable predictive selection of elite strains and support targeted improvement strategies, including mutagenesis, protoplast fusion, and emerging genome-editing approaches. Importantly, these strategies have enhanced antagonistic activity not only against fungal pathogens but also against plant-parasitic nematodes and insect pests, expanding the functional spectrum of *Trichoderma*-based products.

Despite these advances, challenges remain in translating laboratory performance into consistent field efficacy. Environmental variability, formulation stability, and regulatory constraints continue to limit widespread adoption. Addressing these challenges will require tighter integration of genomics, formulation technology, and applied field validation. Genome-guided strain selection, combined with innovative delivery systems and microbial consortia, offers a promising pathway toward more predictable and resilient biocontrol solutions.

In summary, *Trichoderma* has progressed from a taxonomically complex soil fungus to a genomics-enabled biotechnological platform. By integrating molecular systematics, functional genomics, and strain engineering, future research can unlock its full potential for sustainable agriculture, biological pest management, and industrial biotechnology. This integrative approach will be critical for developing next-generation *Trichoderma*-based products with improved efficacy, stability, and regulatory acceptance.

## Data Availability

No datasets were generated or analyzed during the current study.

## References

[CR1] Adnan A, Ma X, Olsson S, Wang J, Liu G (2022) Promoter regulation and genetic engineering strategies for enhanced cellulase expression in *Trichoderma reesei*. Microbiol Res 259:127011 35339938 10.1016/j.micres.2022.127011

[CR2] Adomako J, Larbi-Koranteng S, Twum S, Danso Y (2025) Screening of indigenous *Trichoderma* isolates for their nematocidal potential against root-knot nematodes (*Meloidogyne* spp.) attacking tomatoes (*Solanum lycopersicum* L.). J Hort Res 33:67–76. 10.2478/johr-2025-0001

[CR3] Ahmed AM, El -Katatny MH (2007) Entomopathogenic fungi as biopesticides against the Egyptian cotton leafworm, *Spodoptera littoralis*: between biocontrol-promise and immune-limitation. J Egypt Soc Toxicol 37:39–51

[CR4] Atta NA, Fahmi AI, Abdel-Lateif KS, Nagaty HH, Abd El-Ghany EM (2025) Multilocus identification and genetic enhancement of *Trichoderma* spp. for entomopathogenic activity against *Spodoptera littoralis*. Microb Cell Fact 24:202. 10.1186/s12934-025-02834-6 40936078 10.1186/s12934-025-02834-6 PMC12427115

[CR5] Ávila-Oviedo JL, Chávez-Avilés MN (2026) Ecological versatility and biocontrol mechanisms of *Trichoderma* spp.: toward sustainable agriculture. Discov Appl Sci 8:230. 10.1007/s42452-026-08253-5

[CR6] Ayaz M, Li C-H, Ali Q, Zhao W, Chi Y-K, Shafiq M, Ali F, Yu X-Y, Yu Q, Zhao J-T, Yu J-W, Qi R-D, Huang W-K (2023) Bacterial and fungal biocontrol agents for plant disease protection: journey from lab to field, current status, challenges, and global perspectives. Molecules 28(18):6735 37764510 10.3390/molecules28186735 PMC10537577

[CR7] Baidya S, Manandhar S, Manandhar C (2023) Study on some species of *Trichoderma* for the management of root knot nematode (*Meloidogyne* spp.) in tomato. J Plant Prot Res 8(1):49–59. 10.3126/jpps.v8i1.56447

[CR8] Benítez T, Rincín AM, Limón MC, Codón AC (2004) Biocontrol mechanisms of *Trichoderma* strains. Int Microbiol 7:249–260 15666245

[CR9] Bint-e-Zahira S, Khalid AN, Yousaf N, Iqbal M, Anwar T, Qureshi H, Salmen SH, Ansari MJ (2024) Exploring *Trichoderma* species in industrial wastewater: morphological and molecular insights from isolates. Life 14:750. 10.3390/life14060750 38929733 10.3390/life14060750 PMC11204433

[CR10] Bissett J (1984) A revision of the genus *Trichoderma*. II. Infrageneric classification. Can J Bot 62:914–923

[CR11] Bissett J (1991a) A revision of the genus Trichoderma. II. Infrageneric classification. Can J Bot 69:2357–2372. 10.1139/b91-297

[CR12] Bissett J (1991b) A revision of the genus Trichoderma. III. Section Pachybasium. Can J Bot 69:2373–2417. 10.1139/b91-298

[CR13] Bissett J (1991c) A revision of the genus Trichoderma. IV. Additional notes on section Longibrachiatum. Can J Bot 69(11):2418–2420. 10.1139/b91-299

[CR14] Błaszczyk A, Joachimiak-Lechman K, Sady S, Tański T, Szindler M, Drygała A (2021) Environmental performance of dye-sensitized solar cells based on natural dyes. Sol Energy 215:346–355. 10.1016/j.solener.2020.12.040

[CR15] Chen J, Zhou L, Din IU, Arafat Y, Li Q, Wang J, Wu T, Wu L, Wu H, Qin X, Pokhrel GR, Lin S, Lin W (2021) Antagonistic activity of *Trichoderma* spp. against *Fusarium oxysporum* in rhizosphere of radix pseudostellariae triggers the expression of host defense genes and improves its growth under long-term monoculture system. Front Microbiol 12:579920. 10.3389/fmicb.2021.579920 33790872 10.3389/fmicb.2021.579920 PMC8005620

[CR16] Chen W, Lee MK, Jefcoate C, Kim SC, Chen F, Yu JH (2014) Fungal cytochrome P450 monooxygenases: their distribution, structure, functions, family expansion, and evolutionary origin. Genome Biol Evol 6:1620–1634. 10.1093/gbe/evu132 24966179 10.1093/gbe/evu132 PMC4122930

[CR17] Chen X, Lu Y, Liu X, Gu Y, Li F (2025) *Trichoderma*: dual roles in biocontrol and plant growth promotion. Microorganisms 13:1840. 10.3390/microorganisms13081840 40871343 10.3390/microorganisms13081840 PMC12388180

[CR18] Contreras-Soto MB, Tovar-Pedraza JM, Solano-Báez AR, Bayardo-Rosales H, Márquez-Licona G (2025) Biocontrol strategies against plant-parasitic nematodes using *Trichoderma* spp.: mechanisms, applications, and management perspectives. J Fungi 11:517. 10.3390/jof11070517 10.3390/jof11070517 PMC12300834 40985428

[CR19] Di Lelio I, Coppola M, Comite E, Molisso D, Lorito M, Woo SL, Pennacchio F, Rao R, Digilio MC (2021) Temperature Differentially Influences the Capacity of Trichoderma Species to Induce Plant Defense Responses in Tomato Against Insect Pests. Front. Plant Sci. 12:678830. 10.3389/fpls.2021.678830 10.3389/fpls.2021.678830 PMC8221184 34177994

[CR20] Dolatabad HK, Javan-Nikkhah M, Safari M, Golafaie TP (2019) Effects of protoplast fusion on the antifungal activity of *Trichoderma* strains and their molecular characterization. Arch Phytopathol Plant Prot 52:1255–1275. 10.1080/03235408.2019.1703482

[CR21] Druzhinina I, Seidl-Seiboth V, Herrera-Estrella A, Horwitz BA, Kenerley CM, Monte E, Mukherjee PK, Zeilinger S, Grigoriev IV, Kubicek CP (2011) Trichoderma: the genomics of opportunistic success. Nat Rev Microbiol 9:749–759. 10.1038/nrmicro2637 21921934 10.1038/nrmicro2637

[CR22] Druzhinina IS, Chenthamara K, Zhang J, Atanasova L, Yang D, Miao Y et al (2018) Massive lateral transfer of genes encoding plant cell wall-degrading enzymes to the mycoparasitic fungus Trichoderma from its plant-associated hosts. PLoS Genet 14(4): e1007322. 10.1371/journal.pgen.1007322 10.1371/journal.pgen.1007322 PMC5908196 29630596

[CR23] Druzhinina IS, Kopchinskiy AG, KomoÅ„ M, Bissett J, Szakacs G, Kubicek CP (2005) An oligonucleotide barcode for species identification in *Trichoderma* and *Hypocrea*. Fungal Genet Biol 42:813–828 16154784 10.1016/j.fgb.2005.06.007

[CR24] El-Nagdi WMA, Youssef MMA, El-Khair HA, Abdel-Gawad MM (2019) Effect of certain organic amendments and *Trichoderma* species on the root-knot nematode, *Meloidogyne incognita*, infecting pea (*Pisum sativum* L.) plants. Egypt J Biol Pest Control 29:75. 10.1186/s41938-019-0182-0

[CR25] El-Sharkawy HHA, Abbas MS, Soliman AS. Ibrahim SA, El-Nady (2024) IAI. Synergy between bio-control agents to trigger the defensive responses against Rhizoctonia root rot and enhance the growth, yield, and physiological and anatomical traits of pea plants. J Plant Pathol. 178:104939. 10.1016/j.pestbp.2021.104939

[CR26] El-Sobky MA, Eissa RA, Abdel-Lateif KS, Fahmi AI, El-Zanaty AM, Hassan MM, Elsharkawy MM (2024) Genetic diversity assessment of *Trichoderma* spp. isolated from various Egyptian locations using its gene sequencing marker, rep-PCR, and their cellulolytic activity. Egypt J Biol Pest Control 34:24. 10.1186/s41938-024-00784-6

[CR27] Erayya S, Shukla N, Arzoo K, Kalmesh M, Kumar J (2025) Morphological characterization of *Trichoderma* isolates purified from the rhizosphere soil, collected from copper mining areas of Uttarakhand, India. J Adv Biol Biotechnol 28(8):981–989. 10.9734/jabb/2025/v28i82771

[CR28] Fahmi AI, Al-Talhi AD, Hassan MM (2012) Protoplast fusion enhances antagonistic activity in *Trichoderma* spp. Nat Sci 10(5):100–106

[CR29] Fahmi AI, Eissa RA, El-Halfawi KA, Hamza HA, Helwa MS (2016) Identification of *Trichoderma* spp. by DNA barcode and screening for cellulolytic activity. J Microb Biochem Technol 8:202–209. 10.4172/1948-5948.1000286

[CR30] Gams W, Bissett J (2002) Morphology and classification of *Trichoderma*. Mycol Res 106:131–150

[CR31] Geddes-McAlister J (2022) Systems biology in fungal research. J Fungi 8:478. 10.3390/jof8050478 10.3390/jof8050478 PMC9142880 35628734

[CR32] Gutiérrez-Moreno K, Olguín-Martínez AI, Montoya-Martínez AC, de los Santos-Villalobos S (2025a) *Trichoderma* in sustainable agriculture and the challenges related to its effectiveness. Diversity 17:734. 10.3390/d17100734

[CR33] Gutiérrez-Moreno K, Olguín-Martínez AI, MontoyaMartínez AC, de los SantosVillalobos S (2025b) *Trichoderma* in sustainable agriculture and the challenges related to its effectiveness. Diversity 17:734. 10.3390/d17100734

[CR34] Guzmán-Guzmán P, Etesami H, Santoyo G (2025) *Trichoderma* : a multifunctional agent in plant health and microbiome interactions. BMC Microbiol 25:434. 10.1186/s12866-025-04158-2 40652165 10.1186/s12866-025-04158-2 PMC12255041

[CR35] Guzmán-Guzmán P, Kumar A, de los Santos-Villalobos S, Parra-Cota FI, Orozco-Mosqueda MdC, Fadiji AE, Hyder S, Babalola OO, Santoyo G (2023) *Trichoderma* species: our best fungal allies in the biocontrol of plant diseases—a review. Plants 12:432. 10.3390/plants12030432 36771517 10.3390/plants12030432 PMC9921048

[CR36] Guzmán-Guzmán P, Valencia-Cantero E, Santoyo G (2024) Plant growth-promoting bacteria potentiate antifungal and plant-beneficial responses of *Trichoderma atroviride* by upregulating its effector functions. PLoS 19(3):e0301139. 10.1371/journal.pone.0301139 10.1371/journal.pone.0301139 PMC10959389 38517906

[CR37] Harman G, Howell V, Viterbo A, Chet I, Lorito M (2004) Trichoderma species - Opportunistic, avirulent plant symbionts. Nature Reviews. Microbiology 2:43–56. 10.1038/nrmicro797 10.1038/nrmicro797 15035008

[CR38] Harman G, Khadka R, Doni F, Uphoff N (2021) Benefi ts to plant health and productivity from enhancing plantmicrobial symbionts. Front Plant Sci 11:610065. 10.3389/fpls.2020.610065 10.3389/fpls.2020.610065 PMC8072474 33912198

[CR39] Hasan SA, Abed AZ, Alderawii MM, Abass MH, Alyousuf A, Mustafa AA (2025) First record of *Trichoderma harzianum* as a potent biocontrol agent against red palm weevil *Rhynchophorus ferrugineus* (Olivier 1790) in Iraq. Basrah J Agric Sci 38(Special Issue):387–399. 10.37077/25200860.2025.38.sp.34

[CR40] Hasna MK, Paul NR, Haque MM, Bir SH, Ali A, Chong KP (2025) Biocontrol efficacy of *Trichoderma asperellum* against fusarium wilt in tomato plants by induction of the host defense genes. Discov Plants 2:136. 10.1007/s44372-025-00224-1

[CR41] Hewedy OA, El-zanaty AM, Fahmi AI (2020) Screening and identification of novel cellulolytic *Trichoderma* species from Egyptian habitats. Biotechnologia 101(2):117–133. 10.5114/bta.2020.94771

[CR42] Ismaiel A, Jambhulkar PP, Sinha P, Lakshman DK (2024a) *Trichoderma* : Harzianum clade in soils from Central and South America. J Fungi 10:813. 10.3390/jof10120813 10.3390/jof10120813 PMC11676294 39728309

[CR43] Ismaiel A, Lakshman DK, Jambhulkar PP, Roberts DP (2024b) *Trichoderma* : population structure and genetic diversity of species with high potential for biocontrol and biofertilizer applications. Appl Microbiol 4:875–893. 10.3390/applmicrobiol4020060

[CR44] Jaklitsch WM, Voglmayr H (2015) Biodiversity of *Trichoderma (Hypocreaceae)* in southern Europe. Stud Mycol 80:1–87. 10.1016/j.simyco.2014.11.001 26955191 10.1016/j.simyco.2014.11.001 PMC4779795

[CR45] Kara H (2025) Effect of *Trichoderma **harzianum* Rifai and *Trichoderma **viride* Pers. (Ascomycota: Hypocreales) on demographic parameters of *Myzus**persicae* (Sulzer, 1776) (Hemiptera: Aphididae) feeding on bell pepper plant. Türk Entomol Derg 49(2):175–185. 10.16970/entoted.1572732

[CR46] Khan RAA, Najeeb S, Hussain S, Xie B, Li Y (2020) Bioactive secondary metabolites from *Trichoderma* spp. against phytopathogenic fungi. Microorganisms 8:817. 10.3390/microorganisms8060817 32486107 10.3390/microorganisms8060817 PMC7356054

[CR47] Kubicek CP, Herrera-Estrella A, Seidl-Seiboth V et al (2011) Comparative genome sequence analysis underscores mycoparasitism as the ancestral lifestyle of Trichoderm a. Genome Biol 12: R40. 10.1186/gb-2011-12-4-r40 10.1186/gb-2011-12-4-r40 PMC3218866 21501500

[CR48] Kulik T, Staniszewska P, Wiśniewski P, Treder Z, Przybylski M, Wrońska E, Maździarz M, Krawczyk K, Bilska K, Paukszto Ł, Olszewski J (2025) In-depth comparison of commercial *Trichoderma*-based products: integrative approaches to quantitative analysis, taxonomy and efficacy. Front Microbiol 16:1646394. 10.3389/fmicb.2025.1646394 41048491 10.3389/fmicb.2025.1646394 PMC12488588

[CR49] Kumari R, Kumar V, Arukha AP, Rabbee MF, Ameen F, Koul B (2024) Screening of the Biocontrol Efficacy of Potent Trichoderma Strains against Fusarium oxysporum f.sp. Ciceri and Scelrotium rolfsii Causing Wilt and Collar Rot in Chickpea. Microorganisms 12(7):1280. 10.3390/microorganisms12071280 10.3390/microorganisms12071280 PMC11278996 39065049

[CR50] Lana M, Simón O, Velasco P, Rodríguez VM, Caballero P, Poveda J (2023) First study on the root endophytic fungus *Trichoderma hamatum* as an entomopathogen: development of a fungal bioinsecticide against cotton leafworm (*Spodoptera littoralis*). Microbiol Res 270:127334. 10.1016/j.micres.2023.127334 36804128 10.1016/j.micres.2023.127334

[CR51] Lin X, Heitman J (2005) Chlamydospore formation during hyphal growth in *Cryptococcus neoformans*. Eukaryot Cell 4:1746–1754. 10.1128/EC.4.10.1746-1754.2005 16215181 10.1128/EC.4.10.1746-1754.2005 PMC1265899

[CR52] Marzano M, Gallo A, Altomare C (2013) Improvement of biocontrol efficacy of *Trichoderma harzianum* vs. *Fusarium oxysporum* f. sp. *lycopersici* through UV-induced tolerance to fusaric acid. Biol Control 67:397–408. 10.1016/j.biocontrol.2013.09.008

[CR53] Meena LK, Jambhulkar PP (2024) Determination of chitinase activity of *Trichoderma* isolates on colloidal chitin supplemented medium. Int J Adv Biochem Res 8:406–410. 10.33545/26174693.2024.v8.i11Sf.2915

[CR54] Mondal A, Parvez SS, Bera D, Alam M, Banik A (2026) *Trichoderma* in multitrophic plant–microbe interactions: a pan-genome guided roadmap for resilient physiology and sustainable bio-economy. Plant Physiol Biochem 232:111193. 10.1016/j.plaphy.2026.111193 41802390 10.1016/j.plaphy.2026.111193

[CR55] Monte E (2023) The sophisticated evolution of *Trichoderma* to control insect pests. Proc Natl Acad Sci U S A 120:e2301971120. 10.1073/pnas.2301971120 36913591 10.1073/pnas.2301971120 PMC10041122

[CR56] Mukherjee PK, Horwitz BA, Kenerley CM (2012) Secondary metabolism in *Trichoderma*–a genomic perspective. Microbiol 158:35–45. 10.1099/mic.0.053629-0 10.1099/mic.0.053629-0 21998165

[CR57] Muljowati JS, Oedjijono O, Dewi RS, Mariana A, Chemeltorit P (2025) Exploration and morphological characterization of *Trichoderma* spp from organic waste at TPST Rempoah-Baturraden, Banyumas Regency. E3S Web Conf 609:01003. 10.1051/e3sconf/202560901003

[CR58] Naqvi SAH, Abbas A, Hasnain A, Bilal Z, Hakim F, Shabbir M, Amin A, Iqbal UI (2025) Advancing fungal phylogenetics: integrating modern sequencing, dark taxa discovery, and machine learning. Arch Microbiol 207:192. 10.1007/s00203-025-04392-2 40643763 10.1007/s00203-025-04392-2

[CR59] Ogawa K, Ohara H, Koide T, Toyama N (1989) Interspecific hybridization of *Trichoderma reesei* by protoplast fusion. J Ferment Bioeng 67:207–209. 10.1016/0922-338X(89)90124-4

[CR60] Papzan Z, Kowsari M, Javan-Nikkhah M, Gohari AM, Limón MC (2021) Strain improvement of *Trichoderma* spp. through two-step protoplast fusion for cellulase production enhancement. Can J Microbiol 67(5):406–414. 10.1139/cjm-2020-0438 33226848 10.1139/cjm-2020-0438

[CR61] Peberdy JF (1980) Protoplasts fusion-a tool for genetic manipulation and breeding in industrial microorganisms. Enzyme Microb Technol 2:23–29. 10.1016/0141-0229(80)90004-6

[CR62] Pfordt A, Douanla‑Meli C, Schäfer BC, Schrader G, Tannen E, Chandarana MJ, von Tiedemann A (2025) Phylogenetic analysis of plant‑pathogenic and non‑pathogenic *Trichoderma* isolates on maize from plants, soil, and commercial bio‑products. Appl Environ Microbiol 91(3):e01931‑24. 10.1128/aem.01931-24 10.1128/aem.01931-24 PMC11921352 40013788

[CR63] Poveda J, Velasco P, de Haro A, Johansen TJ, McAlvay AC, Möllers C et al (2021) Agronomic and metabolomic side-effects of a divergent selection for indol-3-ylmethylglucosinolate content in kale (Brassica oleracea var. acephala). Metabolites 11:384. 10.3390/metabo11060384 10.3390/metabo11060384 PMC8231911 34198476

[CR64] Prabavathy V, Mathivanan N, Sagadevan E, Murugesan K, Lalithakumari D (2006) Self-fusion of protoplasts enhances chitinase production and biocontrol activity in *Trichoderma harzianum*. Bioresour Technol 97:2330–2334. 10.1016/j.biortech.2005.10.031 16330207 10.1016/j.biortech.2005.10.031

[CR65] Prismantoro D, Chua K, Teo KW, Chan R, Jefferson TA, Suhaimi NSM, Mispan MS, Miranti M, Doni F (2025) Whole genome sequence data of *Trichoderma yunnanense* strain TM10, a plant growth-promoting fungus and biocontrol agent. Data Brief 58:111283. 10.1016/j.dib.2025.111283 39895666 10.1016/j.dib.2025.111283 PMC11783055

[CR66] Rex JH, Walsh TJ, Sobel JD, Filler SG, Pappas PG, Dismukes WE (2001) *Trichoderma* species [Online]. Available at: http://www.doctorfungus.org/Thefungi/*Trichoderma* . php. Accessed 18 Mar 2013.

[CR67] Rodríguez-Martínez ES, Rios-Velasco C, Sepúlveda-Ahumada DR, Buenrostro-Figueroa JJ, Correia KC, Guigón-López C, Alvarado-González M (2025) *Trichoderma* species from Semiarid regions and their antagonism against the microorganisms that cause pepper wilt. J Fungi 11:174. 10.3390/jof11030174 10.3390/jof11030174 PMC11942951 40137212

[CR68] Rosolen RR, Aono AH, Almeida DA, Ferreira Filho JA, Horta MAC, De Souza AP (2022) Network analysis reveals different cellulose degradation strategies across *Trichoderma harzianum* strains associated with XYR1 and CRE1. Front Genet 13:807243. 10.3389/fgene.2022.807243 35281818 10.3389/fgene.2022.807243 PMC8912865

[CR69] Rosolen RR, Horta MAC, de Azevedo PHC, Da Silva CC, Sforca DA, Goldman CH, de Souza AP (2023) Whole-genome sequencing and comparative genomic analysis of potential biotechnological strains of *Trichoderma harzianum*, *Trichoderma atroviride*, and *Trichoderma reesei*. Mol Genet Genomics 298:735–754. 10.1007/s00438-023-02013-5 37017807 10.1007/s00438-023-02013-5

[CR70] Rostami M, Shahbazi S, Soleimani R, Ghorbani A (2024) Optimizing sustainable control of *Meloidogyne javanica* in tomato plants through gamma radiation-induced mutants of *Trichoderma harzianum* and *Bacillus velezensis*. Sci Rep 14:17774. 10.1038/s41598-024-68365-z 39090171 10.1038/s41598-024-68365-z PMC11294331

[CR71] Saleh MM, Shafeeq AF, Khairi MA (2023) Biological control of *Meloidogyne javanica* by *Pasteuria penetrans* and *Trichoderma harzianum* on tomato plants. Biodiversitas 24:847–851. 10.13057/biodiv/d240221

[CR72] Samuels GJ, Dodd SL, Gams W, Castlebury LA, Petrini O (2002) Trichoderma species associated with the green mold epidemic of commercially grown Agaricus bisporus. Mycologia 94(1):146–170 (PMID: 21156486) 21156486

[CR73] Schalamun M, Schmoll M (2022) *Trichoderma* – genomes and genomics as treasure troves for research towards biology, biotechnology and agriculture. Frontiers in Fungal Biology 3:1002161. 10.3389/ffunb.2022.1002161 37746224 10.3389/ffunb.2022.1002161 PMC10512326

[CR74] Schoch CL, Seifert KA, Huhndorf S, Robert V, Spouge JL, Levesque A, Chen W, Consortium FB (2012) Nuclear ribosomal internal transcribed spacer (ITS) region as a universal DNA barcode marker for Fungi. Proc Natl Acad Sci (USA) 109:6241–6246. 10.1073/pnas.1117018109 22454494 10.1073/pnas.1117018109 PMC3341068

[CR75] Sharma A, Gupta B, Verma S, Pal J, Mukesh M, Akanksha A, Chauhan P (2023) Unveiling the biocontrol potential of *Trichoderma*. Eur J Plant Pathol 154:569–591. 10.1007/s10658-023-02745-5

[CR76] Shoresh M, Harman GE, Mastouri F (2010) Induced systemic resistance and plant responses to fungal biocontrol agents. Annu Rev Phytopathol 48:21–43. 10.1146/annurev-phyto-073009-114450 20192757 10.1146/annurev-phyto-073009-114450

[CR77] Song M, Lin X, Wei X, Zeng Q, Mu C, Zhou X (2024) *Trichoderma viride* improves phosphorus uptake and the growth of *Chloris virgata* under phosphorus-deficient conditions. Front Microbiol 15:1425034. 10.3389/fmicb.2024.1425034 39027109 10.3389/fmicb.2024.1425034 PMC11255847

[CR78] Sood M, Kapoor D, Kumar V, Sheteiwy MS, Ramakrishnan M, Landi M, Araniti F, Sharma A (2020) *Trichoderma* : the “secrets” of a multitalented biocontrol agent. Plants 9(6):762. 10.3390/plants9060762 32570799 10.3390/plants9060762 PMC7355703

[CR79] Sultana AS, Thiruvudainambi S, Masurkar P, Kumar A (2023) Exploration of potent biocontrol agent against the damping off disease of tomato (*Pythium **aphanidematum*) and its *in vitro* management. BF-AIJ 15(5):283–288

[CR80] Sun H, Khan WD, Siddiqui MH, Alamri S, Naeem S, Mehmood T, Tanveer M, Lu Z (2025) Examining the *Trichoderma* sp. and biochar-mediated reduced nickel toxicity in tomato using isotherm adsorption models and plant growth analysis. Environ Technol Innov 40:104503. 10.1016/j.eti.2025.104503

[CR81] Uddin J, Ullah F, Naz I, Ahmad S, Saljoki A, Khan SS, Salim M (2023) Root-knot nematode pathogen suppression in eggplant using antagonistic fungi. Egypt J Biol Pest Control 33:15. 10.1186/s41938-023-00659-2

[CR82] Van Hee S, Segurado Luchsinger AE, Cusumano A, Masschelein J, Jacquemyn H, Lievens B (2025) The plant-beneficial fungus *Trichoderma harzianum* T22 modulates plant metabolism and negatively affects *Nezara viridula*. BMC Plant Biol 25:615. 10.1186/s12870-025-06650-3 40348966 10.1186/s12870-025-06650-3 PMC12065320

[CR83] Woo SL, Hermosa R, Lorito M, Monte E (2023) *Trichoderma* : a multipurpose, plant-beneficial microorganism for eco-sustainable agriculture. Nat Rev Microbiol 21(5):312–326. 10.1038/s41579-022-00819-5 36414835 10.1038/s41579-022-00819-5

[CR84] Xiao Z, Zhao Q, Li W, Gao L, Liu G (2023) Strain improvement of *Trichoderma harzianum* for enhanced biocontrol capacity: strategies and prospects. Front Microbiol 14:1146210. 10.3389/fmicb.2023.1146210 37125207 10.3389/fmicb.2023.1146210 PMC10134904

[CR85] Zhang F, Ma R, Huang Y, Cui Y, Zhou Q, Gu J (2025) Genome mining of the biocontrol agent *Trichoderma afroharzianum* unearths a key gene in the biosynthesis of anti-fungal volatile sesquiterpenoids. Catalysts 15:341. 10.3390/catal15040341

[CR86] Zhao R, Chen KY, Mao LJ, Zhang CL (2024) Eleven new species of *Trichoderma* (Hypocreaceae, Hypocreales) from China. Mycology 16(1):180–209. 10.1080/21501203.2024.2330400 40083403 10.1080/21501203.2024.2330400 PMC11899217

[CR87] Zheng H, Qiao M, Lv Y, Du X, Zhang K-Q, Yu Z (2021) New species of *Trichoderma* isolated as endophytes and saprobes from southwest China. J Fungi 7:467. 10.3390/jof7060467 10.3390/jof7060467 PMC8230185 34207925

